# Tirzepatide, a dual GIP/GLP-1 receptor co-agonist for the treatment of type 2 diabetes with unmatched effectiveness regrading glycaemic control and body weight reduction

**DOI:** 10.1186/s12933-022-01604-7

**Published:** 2022-09-01

**Authors:** Michael A. Nauck, David A. D‘Alessio

**Affiliations:** 1 Diabetes, Endocrinology and Metabolism Section, Medical Department I, Katholisches Klinikum Bochum gGmbH, St. Josef-Hospital, Ruhr-University Bochum, Gudrunstr. 56, 44791 Bochum, Germany; 2 Division of Endocrinology and Metabolism, Department of Medicine, Duke University Medical Center, Durham, NC 27701 USA

**Keywords:** GLP-1 receptor agonists, GIP/GLP-1 receptor co-agonists, Glycemic control, HbA_1c_, Body weight, Type 2 diabetes

## Abstract

Tirzepatide is the first dual GIP/GLP-1 receptor co-agonist approved for the treatment of type 2 diabetes in the USA, Europe, and the UAE. Tirzepatide is an acylated peptide engineered to activate the GIP and GLP-1 receptors, key mediators of insulin secretion that are also expressed in regions of the brain that regulate food intake. Five clinical trials in type 2-diabetic subjects (SURPASS 1–5) have shown that tirzepatide at 5–15 mg per week reduces both HbA_1c_ (1.24 to 2.58%) and body weight (5.4–11.7 kg) by amounts unprecedented for a single agent. A sizable proportion of patients (23.0 to 62.4%) reached an HbA_1c_ of < 5.7% (which is the upper limit of the normal range indicating normoglycaemia), and 20.7 to 68.4% lost more than 10% of their baseline body weight. Tirzepatide was significantly more effective in reducing HbA_1c_ and body weight than the selective GLP-1 RA semaglutide (1.0 mg per week), and titrated basal insulin. Adverse events related to tirzepatide were similar to what has been reported for selective GLP-1RA, mainly nausea, vomiting, diarrhoea, and constipation, that were more common at higher doses. Cardiovascular events have been adjudicated across the whole study program, and MACE-4 (nonfatal myocardial infarction, non-fatal stroke, cardiovascular death and hospital admission for angina) events tended to be reduced over up to a 2 year-period, albeit with low numbers of events. For none of the cardiovascular events analysed (MACE-4, or its components) was a hazard ratio > 1.0 vs. pooled comparators found in a meta-analysis covering the whole clinical trial program, and the upper bounds of the confidence intervals for MACE were < 1.3, fulfilling conventional definitions of cardiovascular safety. Tirzepatide was found to improve insulin sensitivity and insulin secretory responses to a greater extent than semaglutide, and this was associated with lower prandial insulin and glucagon concentrations. Both drugs caused similar reductions in appetite, although tirzepatide caused greater weight loss. While the clinical effects of tirzepatide have been very encouraging, important questions remain as to the mechanism of action. While GIP reduces food intake and body weight in rodents, these effects have not been demonstrated in humans. Moreover, it remains to be shown that GIPR agonism can improve insulin secretion in type 2 diabetic patients who have been noted in previous studies to be unresponsive to GIP. Certainly, the apparent advantage of tirzepatide, a dual incretin agonist, over GLP-1RA will spark renewed interest in the therapeutic potential of GIP in type 2 diabetes, obesity and related co-morbidities.

**Supplementary Information:**

The online version contains supplementary material available at 10.1186/s12933-022-01604-7.

## The concept of co-agonists: molecules with effects on more than one peptide hormone receptor

The redundancy of the system to regulate food intake and body weight led to suggestions that multiple signaling pathways would need to be targeted for effective medical treatment of obesity [1]. Proof-of-concept studies were reported using simultaneous administration of peptides, e.g. leptin and amylin, to cause greater weight loss in rodent models [2]. In 2009, Day and colleagues reported the development of novel peptides engineered to agonize both the GLP-1 and glucagon receptors (GLP-1R and GCGR; [3]). The rationale for these agents was that combining the satiety effects of GLP-1 signaling with purported effects of glucagon to increase energy expenditure would lead to a more effective weight loss agent. In fact, this was demonstrated quite convincingly in obese mice, with some variability in effect depending on the relative balance of potency at the GLP-1R and GCGR. Of perhaps greater importance was the establishment of the process of iterative chemical refinement as a means of modifying the amino acid sequence of a known peptide to add agonism for multiple receptors [4]. Using this model an obvious strategy was to combine activity for GLP-1 with that of glucose-dependent insulinotropic polypeptide (GIP) to treat diabetes. Together GLP-1 and GIP account for the bulk of the incretin effect, a physiologic system evolved to augment insulin secretion following the ingestion of nutrients [5]. Both the GLP-1R and the GIP receptor (GIPR) are expressed on pancreatic β-cells and activation of these in the context of even modest elevations of blood glucose potently stimulate insulin secretion [6]. Activation of the GLP-1R lowers blood glucose in persons with type 2 diabetes while the GIPR is much less effective for this [7, 8]. Nonetheless, an engineered peptide with activity at both the GIPR and GLP-1R was more effective at reducing body weight and blood glucose in obese mice than a selective GLP-1R agonist (GLP-1RA) [9]. This compound was also superior to a GLP-1RA for stimulating insulin secretion in non-human primates, and reduced blood glucose in humans with diabetes. While the insulinotropic properties of a GIPR/GLP-1R co-agonist were expected, the effects on food intake and weight loss were not. However, recent work in rodents has demonstrated that the GIPR is expressed on neurons in the arcuate nucleus and other parts of the hypothalamus [10], and their activation reduces food intake and body weight [11], in particular when co-administered with GLP-1 [12]. This initial work, and other studies, heralded the development of a number of different multi-receptor peptides that have become the latest advances in therapeutics of diabetes and obesity.

### Methodological details

**Fig. 1:**
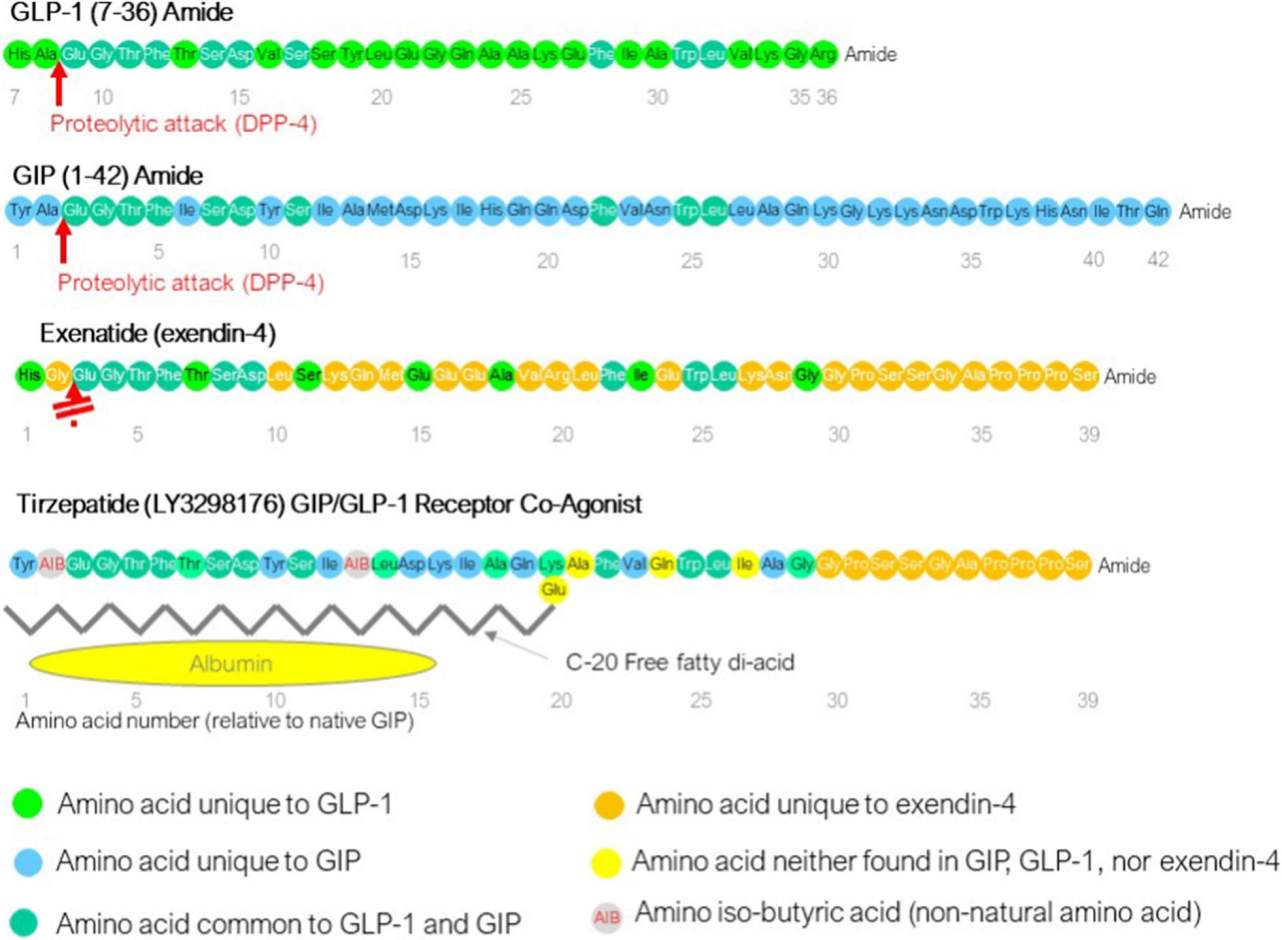
Amino acid sequences of the incretin hormones GLP-1 (glucagon-like peptide-1) and GIP (glucose-dependent insulinotropic polypeptide), the GLP-1 receptor agonist exenatide, and tirzepatide, a GIP/GLP-1 receptor co-agonist. Colours indicate amino acids in the peptide sequence of tirzepatide which correspond to amino acids in the original primary structure of GLP-1 (green), GIP (blue), shared by both GLP-1 and GIP (blue-green), exenatide (orange). Amino acids not related to any of the parent peptides are shown in yellow. Amino-iso-butyric acid (AIB), a non-natural amino acid, is shown in grey with red letters. The primary amino acid sequence of tirzepatide has been taken from [13]; the sequences for human GIP, mammalian GLP-1, and exenatide for comparison are from [22–24]

**Fig. 2:**
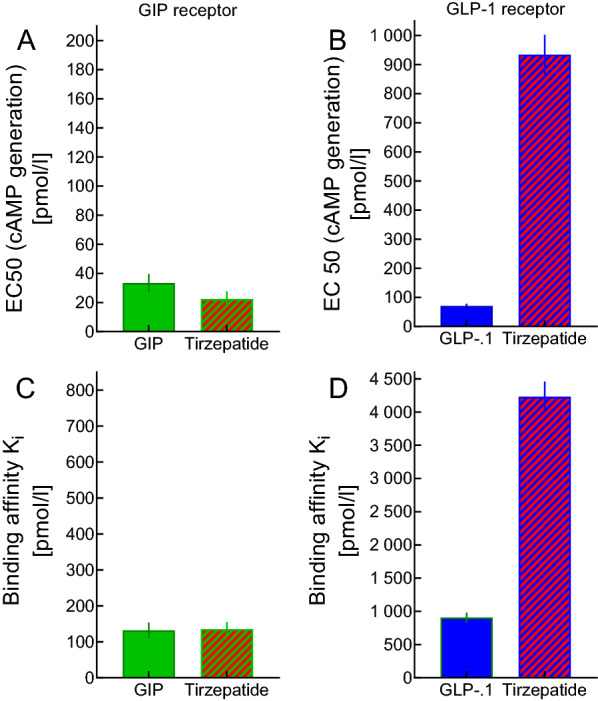
Binding affinity of GIP, GLP-1, and the dual (GIP and GLP-1 receptor) co-agonist tirzepatide (formerly named LY3298176) to human embryonic kidney (HEK 293) cells transfected with human GIP and GLP-1 receptors, and potency in stimulating cyclic adenosine mono-phosphate (cAMP) accumulation. Data have been taken from Coskun et al. 2018 [13] and are expressed as EC50 (effective concentration resulting in half-maximal stimulation) or K_i_ (inhibitory constant, leading to half-maximal displacement of tracer)

**Fig. 3:**
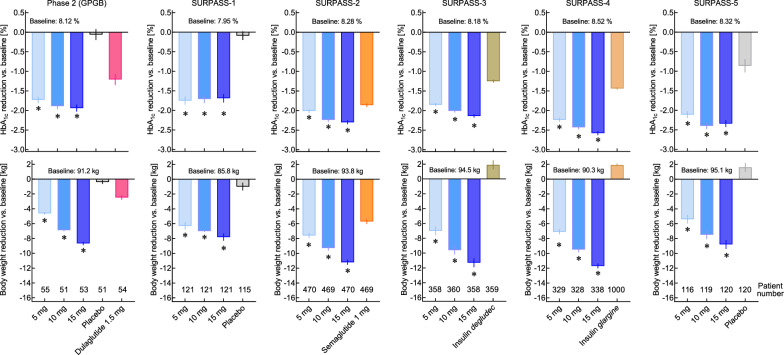
Efficacy of tirzepatide in phase 2 (GPGB; [14]) and phase 3 (SURPASS-1 to 5; [15–19]) clinical trials, all according to the treatment estimand (SURPASS 1–5) or by Bayesian modified intention-to-treat analysis without considering data acquired post-rescue (GPWB). The upper row of panels depicts effects on HbA_1c_ (reduction vs. baseline). The lower row of panels shows effects on body weight (reduction vs. baseline). Asterisks (_*_) indicate a significant difference (p < 0.05) vs. the respective comparator. Comparators were placebo and dulaglutide (1.5 mg per week) in phase 2 (GPGB), placebo (grey; SURPASS-1 and -5), semaglutide 1.0 mg (orange; SURPASS-2), basal insulin *degludec* (darker brown; SURPASS-3) and basal insulin *glargine* U100 (lighter brown; SURPASS-4). Patient numbers per arm are presented in the bottom of the lower panels

**Fig. 4:**
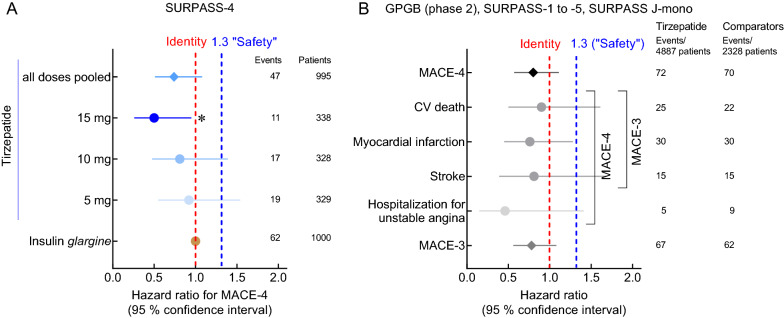
Effects of tirzepatide on adjudicated cardiovascular events in SURPASS-4 [18] (**A**), a clinical trial recruiting subjects at high risk for cardiovascular events, and across the clinical trial program (phases 2 and 3) for tirzepatide [20] (**B**). MACE: Major adverse cardiovascular events. The line of identity (red, dashed) indicates an equal risk for CV events for tirzepatide and comparator(s). The blue, dashed line marks a hazard ratio of 1.3. An upper bound of the confidence interval for composite endpoints (MACE-3 and MACE-4, but not for individual endpoints) below 1.3 conventionally is interpreted as indicating a cardiovascular risk, which is not significantly elevated in comparison to the comparator(s). Comparators include placebo, insulin *degludec*, insulin *glargine*, dulaglutide 1.5 mg/week, and *semaglutide* 1.0 mg/week (GLP-1 receptor agonists). The numbers of patients at risk and the numbers of events observed are presented. The asterisk indicates a significant difference indirectly inferred from the 95% confidence interval completely being below the line of identity (= 1)

This review is based primarily on information from preclinical studies in animal models and clinical trials in humans; a secondary analysis of the trial data has been included to highlight specific points. For this purpose, a PubMed literature search was performed using EndNote X7.1 (www.endnote.com), using the search terms “tirzepatide”, “LY3298176 “, "dual GIP/GLP-1” and “GIP/GLP-1 receptor co-agonist”. Data in Tables 1 and 2 were taken from publications of phase 2 (GPWB) and 3 (SURPASS 1–5) trials. Data presented as Fig. 1 (molecular structure of tirzepatide and its relationship to GIP, GLP-1, and exendin-4) have been adopted from publications quoted in the respective legend. Data presented as Fig. 2 were taken from Coscun et al. 2018 [13]. Data presented as Fig. 3 have been taken from publications of phase 2 (GPWB) [14] and phase 3 (SURPASS 1–5) trials [15–19], strictly presenting the treatment estimand (or, in the case of GPWB, a Bayesian analysis according to a modified intention-to-treat approach excluding data gathered post-rescue, which is most similar to the treatment estimand, but, however, does not exclude data after treatment discontinuation). Data presented as Fig. 4 are redrawn from the SURPASS-4 publication [18] and from a publication summarizing cardiovascular events across the tirzepatide clinical trial program [20]. Dose–response relationships presented as Additional file 1: Figures  S1, S2, S3 have been re-analyzed for the present manuscript from published data of the SURPASS 1–5 trials [15–19], uniformly extracting data according to the efficacy estimand (excluding data after drug discontinuation or rescue therapy), i.e. in patients precisely on their randomized treatment, as appropriate for the analysis of a dose–response relationship. For the 5, 10, and 15 mg per week doses, weighted means and pooled standard errors of the mean (derived from pooled standard deviations and the number of patients in each trial) were calculated and displayed and compared by analysis of variance. This analysis assumes comparable dose response relationships across the distinct cohorts in the SURPASS studies. The degree of homogeneity was estimated by calculating q and I^2^ statistics. For categorial variables (proportion of patients with target achievement and adverse events), 95% confidence intervals were calculated and displayed and compared by χ^2^ tests.

**Table 1:** Baseline characteristics of patients with type 2 diabetes participating in clinical trials (phase 2 and 3) with the GIP/GLP-1 receptor co-agonist tirzepatide

Parameter	Unit	Phase 2 (GPGB) [14]	SURPASS-1 [15]	SURPASS-2 [16]	SURPASS-3 [17]	SURPASS-4 [18]	SURPASS-5 [19]
Comparator(s)		Placebo, dulaglutide 1.5 mg	Placebo	Semaglutide 1 mg	Insulin *degludec*	Insulin *glargine*	Placebo
Study duration	Weeks	26	40	40	52	52/104*	
Overall patient number		316**	478	1878	2874	1995	475
Background medication			None (monotherapy^†^)	Metformin	Metformin ± SGLT-2 inhibitor	Metformin ± SGLT-2 inhibitor or sulfonylurea (alone or in combination)	Insulin *glargine * ± metformin
Age	Years	57.2	54.1	56.6	57.4	63.6	60.7
Female	%	47.3	48	53	56	38	44
Duration of diabetes	years	8.7	4.7	8.6	8.4	10.5	13.3
HbA_1c_	%	8.12	7.94	8.28	8.17	8.52	8.32
Fasting plasma glucose	mmol/l	9.4	8.5	9.6	9.4	9.5	9.0
Body-mass-index	kg/m^2^	32.5	31.9	34.2	33.5	32.6	33.4
Receiving metformin	%	90.5	–	100	100	95	82.5
Receiving SGLT-2 inhibitor	%	–	–	–	32	25	–
Receiving sulfonylurea	%	–	–	–	–	54	–
eGFR	ml/min per 1.73 m^2^	92.7	94.1	96.0	94.1	81.3	85.5

**Table 2:** “Gastro-intestinal” adverse events reported in clinical trials comparing tirzepatide (5, 10, and 15 mg pre week) with selective GLP-1 receptor agonists (dulaglutide 1.5 mg/week and semaglutide 1.0 mg per week)

Study	Phase 2 (GPGB), 26 weeks [14]				SURPASS-2, 40 weeks [16]			
Agent	Tirzepatide			Dulaglutide	Tirzepatide			Semaglutide
Dose	5 mg	10 mg	15 mg	1.5 mg	5 mg	10 mg	15 mg	1 mg
Patient numbers per arm	55	51	53	54	470	469	470	469
Effectiveness								
HbA_1c_ reduction vs. baseline [%]*	−1.6	−2.0	−2.4	−1.1	−2.0	−2.2	−2.3	−1.9
Body weight reduction vs. baseline [kg]^†^	−4.8	−8.7	−11.3	−2.7	−7.8	−10.3	−12.4	−7.8
Adverse events								
Nausea	20.0	21.6	39.6	29.6	17.4	19.2	22.1	17.9
Vomiting	7.3	15.7	26.4	9.3	5.7	8.5	9.8	8.3
Diarrhoea	23.6	23.5	32.1	16.7	13.2	16.4	13.8	11.5
Constipation	3.6	11.8	3.8	5.6	6.8	4.5	4.5	5.8
Any “gastro-intestinal” adverse event	32.7	51.0	66.0	42.6	40.0	46.1	44.9	41.2
Adverse event leading to treatment discontinuation	9.1	5.9	24.5	11.1	6.0	8.5	8.5	4.1

### Tirzepatide (LY3298176): pharmacologic development of a unimolecular dual GLP-1R/GIPR co-agonist

Tirzepatide was one of several single molecule GIPR and GLP-1R agonists developed after the early work in multi-receptor peptides. It is a 39 amino acid linear peptide, similar in size to GIP and GLP-1, which are related hormones both belonging to the secretin family of gut peptides. The starting amino acid sequence was that of human GIP and tirzepatide retains 9 homologous amino acids from this peptide as well as 10 amino acids shared by GIP and GLP-1. Four amino acids correspond to the same position in the GLP-1 molecule, and 10 amino-terminal amino acids are identical to those in the sequence of exendin-4 (a peptide from the saliva of a lizard called *Heloderma suspectum*, later becoming exenatide, the first GLP-1 RA [21]). In addition, amino-iso-butyric acid residues are found in positions 2 (a recognition site for dipeptidyl peptidase-4) and 13, and three amino acid positions are unique to tirzepatide. An (AEEA)2-gamma Glu-C20 diacid is attached to the lysine residue in position 20, which promotes binding to albumin, and is essential in promoting a long duration of action (like in liraglutide and semaglutide as well as in long-acting insulin analogues *degludec* and *icodec*). The sequence of tirzepatide and the origin of each amino acid within the sequence is shown as Fig. 1.

In pancreatic ß-cells, tirzepatide stimulates cAMP generation much like a combination of GIP and GLP-1 [13]. The dose–response relationship for cAMP production in human embryonic kidney cells (HEK293) transfected with human incretin receptors indicates similar activation of GIPR receptors as native GIP, with lower potency on GLP-1R compared to endogenous GLP-1 [13]. Tracer displacement studies as well as cAMP generation studies show binding to and stimulation of GIP receptors similar to native GIP, whereas binding and effects at the GLP-1 receptor are less than native GLP-1 [13]. In keeping with the data in transfected cells, cAMP production in differentiated human adipocytes with tirzepatide follows the same dose–response relationship as for human GIP [13]. Overall tirzepatide seems to preferentially activate GIP receptors over GLP-1 receptors in isolated cell systems with high-grade expression of GIP and GLP-1 receptors. Structural prerequisites explaining receptor bifunctionality as well as the preferential binding to and activation of GIP relative to GLP-1 receptors have been extensively studied [25]. Concentrations of tirzepatide, GIP and GLP-1 leading to half maximal tracer displacement (binding affinity) and to half-maximal cAMP generation reported in Coskun et al. 2018 [13] are displayed in Fig. 2. In murine pancreatic islets lacking GLP-1 receptors, tirzepatide-stimulated insulin secretion was completely blocked by a GIPR antagonist, while in islets from GIP receptor *knock-out* mice, tirzepatide-stimulated insulin secretion is completely blocked by GLP-1R antagonism [13]. In mice with diet-induced obesity, tirzepatide reduced both food intake and body weight significantly better than did semaglutide, the most effective selective GLP-1 RA available [13].

In human subjects, international phase 1 studies indicated a linear relationship between the dose of tirzepatide and peak circulating concentrations (C_max_), a time from injection to C_max_ of 1–2 days, and an elimination half-life of approximately 5 days [13]. In patients with type 2 diabetes, over 29 days, body weight was reduced by up to 3 kg, fasting plasma glucose was reduced by up to 3 mmol/l, plasma glucose following an oral glucose load was dose-dependently reduced, while insulin secretory responses were dose-dependently increased. Similar pharmacokinetic and initial pharmacodynamic data were collected in Japanese subjects with type 2 diabetes [26]. Neither renal functional impairment [27] nor compromised liver function [28] change the pharmacokinetic behavior of tirzepatide. Based on these findings, the dosage of tirzepatide will likely not have to be changed in chronic kidney disease or with reduced liver function, but dedicated clinical studies in such patients are not yet available.

### Tirzepatide phase 2 study in type 2 diabetes: optimization of initial up-titration schedule and definition of effective and tolerable dose range

In the primary phase 2 trial [14], doses of tirzepatide from 1 to 15 mg per week were compared to placebo and the selective GLP-1 receptor agonist dulaglutide, 1.5 mg per week. Baseline characteristics of type 2 diabetes subjects are shown as part of Table 1. Tirzepatide at doses of 5, 10, and 15 mg was more effective than dulaglutide (1.5 mg) in reducing HbA_1c_ (by up to 2.4% from a baseline of 8.12%). Notably, 30.2% of the patients treated with 15 mg per week reached an HbA_1c_ < 5.7% (corresponding to the normal range in healthy subjects; Fig. 3) [14]. Likewise, tirzepatide at doses of 5, 10, and 15 mg was more effective than dulaglutide (1.5 mg) in reducing body weight (by up to 11.3 kg from a baseline of 91.2 kg; Fig. 3)). Notably, 24.5% of patients treated with 15 mg per week reached a body weight reduction of ≥ 15% [14]. Nausea, diarrhoea, and vomiting were identified as the most prominent adverse events (Table 2). Since with the 15 mg per week dose of tirzepatide, 18 out of 53 (34.0%) subjects in the initial phase 2 trial discontinued treatment (13 or 24.5% because of adverse events), slower and more cautious initial up-titration schedules were the dedicated focus of a second phase 2 trial [29], which resulted in a much-reduced discontinuation rate (e.g., 1 out of 28 or 3.6% randomized to tirzepatide 15 mg discontinued because of adverse events). Essential elements of this better tolerated regime of introducing tirzepatide treatment was carried on into phase 3 studies.

### The SURPASS program in type 2 diabetes: proof of effectiveness and of safety/tolerability

The pivotal clinical trial program designed to prove therapeutic effectiveness (glycaemic control and body weight reduction) and safety/tolerability for the treatment of patients with type 2 diabetes has been named the SURPASS program, encompassing trials SURPASS 1–6, SURPASS-J mono and combo, respectively, in Japanese populations, SURPASS-AP in Asian-Pacific patients, and SURPASS CVOT, a dedicated cardiovascular outcomes trial which is ongoing and will report in 2024 [30]. These trials share common features: Tirzepatide treatment is studied at three final doses (5, 10, 15 mg per week), initiating treatment at 2.5 mg, and increasing the dose every 4 weeks. Some SURPASS trials compare tirzepatide to placebo treatment, others use active comparators like semaglutide, a selective GLP-1 receptor agonist, or the basal insulin preparations, insulins *degludec* or *glargine* (see Table 1). SURPASS-CVOT has a different design from the effectiveness/safety/tolerability trials in that it attempts to reach the maximum tolerated dose in every patient, and because it is a two-arm study, using dulaglutide (1.5 mg per week or highest tolerated dose) as comparator [30].

Across the doses ranging from 5 to 15 mg of tirzepatide per week, HbA_1c_ was reduced in SURPASS 1–5 by between 1.69 to 2.58% (Fig. 3), with approximately 24–30 weeks of treatment to reach a new plateau of HbA_1c_ and fasting plasma glucose [15–19]. Among subjects treated with tirzepatide, 81.0–92.9% reached an HbA_1c_ < 7.0%, 66.0–86.0% reached an HbA_1c_ ≤ 6.5%, and 23.0–62.4% reached an HbA_1c_ < 5.7%; the latter corresponding to an accepted correlate of normal glucose tolerance [15–19]. Fasting plasma glucose was reduced in SURPASS 1–5 by between 2.4 and 3.5 mmol/l [15–19]. Across the doses ranging from 5 to 15 mg of tirzepatide per week, body weight was reduced in SURPASS 1–5 by between 5.4 to 11.7 kg (Fig. 3) [15–19]. Remarkably, a plateau was not reached in studies with a duration shorter than 52 weeks [15, 16, 19], and it may take more than a year to achieve a new steady-state body weight after initiating tirzepatide treatment [18].

Tirzepatide at all three doses proved to be significantly more effective than placebo [15, 19], both with respect to parameters of glycaemic control and body weight reduction (Fig. 3). More remarkably, tirzepatide was significantly more efficacious compared to titrated basal insulins *degludec* [17] and *glargine* [18] (Fig. 3). In these trials, tirzepatide at higher doses was at least as effective as basal insulin preparations in controlling fasting plasma glucose. In previous studies of GLP-1RAs, only semaglutide [31], the most potent compound in this class [32, 33], had a similar effect. Predictably, post-prandial glycaemic excursions were better controlled with tirzepatide than with basal insulin [17, 18]. The most remarkable finding, however, is the substantially better efficacy (regarding both HbA_1c_ and body weight reductions) when compared to 1.0 mg semaglutide per week (the standard dose used in most type 2 diabetes trials [34]) (Fig. 3). It is reasonable to infer from this treatment difference that GIPR agonism contributed significantly to the overall effectiveness of tirzepatide.

For a closer look into the dose–response relationships comparing 5, 10, and 15 mg per week of tirzepatide, we refer to a meta-analysis of results reported from SURPASS-1–5 (Additional file 1: Figures. S1, S2, S3). There were significant dose-dependent reductions of HbA_1c_ and body weight from the pretreatment baseline with each dose step from 5 to 15 mg of tirzepatide. This dose-dependency was also seen with fasting glucose, although the trend did not reach statistical significance (Additional file 1: Figure. S1). Similar dose proportionality was seen for HbA_1c_ targets, with higher doses leading to an increased percentage of patients achieving values < 7.0%, ≤ 0.6.5%, or ≤ 0.5.7% (Additional file 1: Figure S2). Dose–response relationships were steepest for the HbA_1c_ ≤ 0.57% target, and shallowest for the HbA_1c_ < 7.0% target. Despite the overall robust treatment effects, it is remarkable that 11.1% of treated subjects did not reach this standard glycemic target even with the highest dose of tirzepatide (compare Additional file 1: Figure S2, details not shown).

The relative reductions in HbA_1c_ and body weight observed with doses of 5, 10 and 15 mg per week of tirzepatide were similar among the 5 SURPASS trials (Fig. 3). Accordingly, post hoc analyses of the SURPASS clinical trial program indicate that the reduction in HbA_1c_ is independent of age [35], duration of diabetes [36], or baseline HbA_1c_ ≤ vs. > 8.5% [37]—the latter results showing meaningful reductions in both subgroups, but with higher baseline HbA_1c_ predicting greater reductions. With respect to body weight, a higher baseline body-mass-index is a predictor of absolute weight reduction, albeit with substantial body weight loss even in those with a BMI < 27 kg/m^2^ [38]. Female vs. male sex does not influence body weight reduction with tirzepatide treatment [39]. Another notable finding from the SURPASS trials is a significant correlation between weight reduction achieved with tirzepatide and the reduction in HbA_1c_, indicating that greater weight loss in the range achievable with tirzepatide has a substantial impact on glycemic control [40], as shown for most but not all the SURPASS trials and doses tested (it was not shown for SURPASS 1 and for 10 mg and 15 mg doses in SURPASS-5, i.e. the clinical studies with smaller patient numbers; Table 1; Fig. 3). No such association has been demonstrated for selective GLP-1 RAs, perhaps because the weight loss in these studies was of lesser magnitude. Nevertheless, 6–13% of patients treated with tirzepatide 5 mg per week, 5–12% with 10 mg, and 3–12% with 15 mg did not lose weight in the SURPASS trials [40], indicating a high degree of inter-individual heterogeneity concerning body weight response, as noted for GLP-1 RAs previously.

Nearly 38% of all tirzepatide-treated patients reached an HbA_1c_ < 5.7%, a value considered nondiabetic [41]. This subgroup was characterized by a slightly younger age and duration of diabetes, lower baseline fasting plasma glucose and HbA_1c_, and greater reduction of glycaemic parameters and body weight. Baseline body-mass-index was not different from patients that did not reach a normal HbA_1c_. Thus, there is substantial inter-individual variability in treatment effects, with a higher likelihood of response in patients with less advanced type 2 diabetes [41]. Recent statements by expert panels from the American Diabetes Association and the European Association for the Study of Diabetes have concluded that an HbA_1c_ below the diabetic range should not be termed remission when continued medication is necessary [42]. Moreover, it is not yet known whether reducing HbA_1c_ to below 5.7% has any impact on the development and progression of diabetes complications, or life expectancy.

### Cardiovascular risk factors and safety

Like selective GLP-1 receptor agonists, tirzepatide improved a number of cardiovascular risk factors. In blood samples analyzed from the phase 2 (GPGB) study, triglycerides and the constituent, apolipoprotein C-III, were dose-dependently reduced, significantly more than with dulaglutide 1.5 mg per week [43]. LDL cholesterol and its constituent, apolipoprotein B, were reduced with tirzepatide, but not more than with dulaglutide [43]. In general, large triglyceride-rich lipoproteins were reduced as were small low-density lipoproteins, while changes with dulaglutide treatment were not significant [43]. In keeping with these changes, total diacylglycerols were dose-dependently reduced with tirzepatide, more than with dulaglutide, as were total phosphatidylethanolamines and phosphatidylcholines [44]. Circulating lipoprotein lipase (in the absence of heparin injection) was increased by tirzepatide treatment, more than with dulaglutide [43]. Circulating concentrations of branched-chain amino acids (leucine, isoleucine, and valine) and branched-chain ketoacids were dose-dependently reduced with tirzepatide, while dulaglutide had no significant effect [44]. Concentrations of 2-hydroxy-butyric acid were reduced more with tirzepatide than with dulaglutide [44]. In the SURPASS-1 and -2 studies, significant reductions in triglycerides (by 18.5 to 24.8%), in LDL cholesterol (by 5.2 to 12.4%), and in VLDL cholesterol (by 17.5 to 23.7%), as well as a significant increase of HDL cholesterol (by 3.2–7.9%) were confirmed [15, 16]. Changes in triglycerides and VLDL cholesterol clearly exceeded those elicited by semaglutide 1.0 mg/week [16]. Systolic blood pressure was reduced in all SURPASS trials in a dose-dependent manner, by approximately 5–6 mmHg [15–19], more substantially than with semaglutide (by 3.6 mmHg on average) in SURPASS-2 [16]. Inflammatory markers like C-reactive protein (but not interleukin-1) and circulating adhesion molecules like ICAM-1 (but not VCAM-1) were reduced by tirzepatide treatment [45].

The effect of tirzepatide treatment on cardiovascular events has been studied in SURPASS-4, a trial recruiting subjects with known coronary, peripheral arterial, or cerebrovascular disease, or who were at high risk for these, i.e. age > 50 years with either chronic kidney disease and an eGFR of less than 60 ml/min per 1.73 m^2^ or history of congestive heart failure (NY Heart Association Class II or III). In addition, SURPASS-4 had the longest duration of the completed studies phase 3 studies tirzepatide trials (Table 1); SURPASS-CVOT will have a considerably longer duration. Figure 4 shows the results of SURPASS-4 by tirzepatide dose as well as for all doses pooled, compared to insulin *glargine* treatment. No tirzepatide dose numerically increased the risk for cardiovascular events. For subjects treated with the highest dose of tirzepatide (15 mg per week), the risk to develop any major adverse cardiovascular event (MACE-4 composed of myocardial infarction, stroke, hospitalization for angina, and all-cause death) was estimated at 0·50 (0·26–0·95). However, this was based on only 11 events in the tirzepatide 15 mg/week group (and 62 events with insulin glargine treatment; Fig. 4A). In addition to the results from SURPASS 4, which was designed to provide preliminary evidence for the cardiovascular safety of tirzepatide, a separate analysis pooling all the clinical trials with tirzepatide was used to generate a pre-specified estimate of cardiovascular events [20] (Table 1). Relative to comparators, the risk for any of the commonly used cardiovascular end points was estimated to be below 1.0, and for both MACE-4 and MACE-3 (myocardial infarction, stroke, and all-cause death), the upper bounds of the confidence interval calculated around the hazard ratio were decidedly < 1.3 (Fig. 4B). Thus, these data suffice as preliminary evidence of cardiovascular safety for tirzepatide according to US Food and Drug Administration guidance. Results in subgroups defined by sex, age, baseline HbA_1c_, ethnic composition, country of origin, and SGLT-2 inhibitor use at baseline did not differ from overall results. A dedicated clinical trial is being performed to provide definite answers regarding the cardiovascular safety and potential cardiovascular benefits of tirzepatide (SURPASS CVOT; https://ClinicalTrials.gov/ct2/show/NCT04255433) [30]. In this study tirzepatide will be compared to dulaglutide, a selective GLP-1 RA with proven cardiovascular benefits [46].

### Adverse events reported with tirzepatide (*dual GIP/GLP-1 receptor co-agonist)*

In clinical trials employing tirzepatide, nausea, vomiting, diarrhoea, and constipation were the main side effects reported. These “gastrointestinal” side effects appear to be qualitatively similar to those reported in clinical studies with selective GLP-1 RAs, and display some dose-dependency (Additional file 1: Figure S3). Therefore, in quantitative terms, a comparison to selective GLP-1 RAs is of particular interest. Table 2 presents the proportion of patients reporting nausea, vomiting, diarrhoea, constipation, or any gastrointestinal adverse event in studies comparing tirzepatide at 5, 10, or 15 mg per week to dulaglutide (1.5 mg per week) [14] or semaglutide (1 mg/week) [16]. As shown in Table 2, higher doses of tirzepatide are associated with more of these side effects (see also Additional file 1: Figure S3).

However, in both studies, there was a trend for numerically greater effectiveness of tirzepatide (5 mg per week) as compared to dulaglutide (phase 2 study; GPWB) or semaglutide (SURPASS-2), with respect to improving glycaemic control (HbA_1c_) and reducing body weight, but fewer patients reported “gastrointestinal” side effects as compared to those treated with dulaglutide or semaglutide. This may indicate that for a given efficacy, tirzepatide elicits less gastrointestinal adverse events than do selective GLP-1 RAs. Along these lines, GIPR agonism in animal experiments had the ability to reduce GLP-1-induced nausea and vomiting, or an equivalent behaviour [47, 48]. The availability of three doses of tirzepatide provides the flexibility to find an individual optimum relating effectiveness and tolerability.

### Mechanisms of action of tirzepatide (dual GIP/GLP-1 receptor co-agonist), with particular emphasis of differences to selective GLP-1 receptor agonists

Given the impressive results of the tirzepatide clinical trials, understanding pharmacologic mechanisms of action is likely to provide insight into the pathogenesis and correction of diabetes and obesity. Moreover, differentiating a GIP/GLP-1 receptor co-agonist from a selective GLP-1 receptor agonist has implications for the broader scope of multi-receptor targeting in metabolic disease. Are there footprints of GIP receptor agonism in the studies to date? An experiment based on the general protocol of the SURPASS-2 study, but including physiological outcomes, was recently reported. At baseline and after 28 weeks of treatment, Heise et al. performed a hyperinsulinaemic, euglycaemic clamp experiment to estimate insulin sensitivity, followed by a hyperglycaemic clamp (216 mg/dl;12.0 mmol/l) to estimate insulin secretory responses. On a second day of study, plasma glucose, insulin and glucagon responses were recorded following a mixed meal test with concurrent assessment of ratings (visual analogue scales) of hunger, satiety, prospective food consumption, and fullness; energy intake was measured on the occasion of an ad libitum meal [49, 50]. The clinical results of this trial mimicked the primary findings of SURPASS-2 with comparable HbA_1c_ reduction and loss of body weight. Insulin sensitivity as measured by the glucose infusion rate needed to maintain euglycaemia rose by 65.7% with tirzepatide (as compared to 37.5% with semaglutide 1.0 mg). Thus, the rise in insulin sensitivity was 20.5% greater with tirzepatide as compared to semaglutide. Some of this effect is likely due to the difference in body weight reduction (average 11.2 kg with tirzepatide vs. 6.9 kg with semaglutide) [50], and in fact, the improvement in insulin sensitivity was related to the individual degree of body weight reduction. However, the slope of the regression line relating insulin sensitivity to amount of weight loss was significantly steeper for tirzepatide compared to semaglutide [49]. These results suggest that there may be weight-dependent and weight-independent components to the improvement in insulin sensitivity accompanying tirzepatide treatment, and that there is a greater improvement in insulin sensitivity per unit weight loss with tirzepatide as compared to semaglutide [49].

The meal tests indicated a significant reduction in fasting and post-meal plasma glucose concentrations with both tirzepatide and semaglutide compared with placebo treatment, and baseline-subtracted plasma glucose increments did not differ between the two drug treatments [50]. Meal-related insulin secretory responses were significantly smaller with tirzepatide compared to both semaglutide and placebo treatment [50], compatible with the improvements in insulin sensitivity. Meal-related rises in plasma glucagon were smaller with tirzepatide than with semaglutide, and substantially and significantly reduced vs. placebo treatment with both semaglutide and tirzepatide [50]. While appetite ratings and ad libitum energy intake were lower with semaglutide and tirzepatide treatment as compared to placebo, they did not differ significantly between the selective GLP-1 RA and the dual GIP/GLP-1 receptor co-agonist [49]. While the described findings help to explain some aspects of the clinical effectiveness of tirzepatide, it is not possible to discern from this experiment whether the distinct effects of tirzepatide relative to semaglutide are the result of GIPR signaling.

Animal studies have identified major effects of tirzepatide on insulin sensitivity in mice with diet-induced obesity [51]. Since, in the same study, a long-acting GIP also had marked effects on insulin sensitivity, an insulin-sensitizing effect of GIPR activation can be inferred. The insulin-sensitizing effect of tirzepatide appears to be relatively more potent in mice with diet-induced obesity [51] than in human subjects with type 2 diabetes [50]. Potential mechanisms include a reduction in ectopic, e.g. liver, pancreas and skeletal muscle, fat deposition, possibly mediated through preferential storage of triacyl glycerols in subcutaneous adipose tissue depots [52]. Details on the effect of GIP receptor agonism on adipose tissue are extensively discussed in Nauck et al. 2021 [53].

In studies of humans with type 2 diabetes, glycaemic control is generally better with tirzepatide than with selective GLP-1 RAs (dulaglutide [14], semaglutide [16]) coincident with a greater insulin response to glucose [50]. One explanation for these results is that GIPR agonism contributes to enhanced insulin secretion in tirzepatide treated patients. However, this conjecture is contrary to previous studies reporting very meager beta-cell stimulation in type 2 diabetic subjects receiving native GIP [7, 59] (Table 3), a deficit that may be due to specific defects in expression or function of GIP receptors, or to a general impairment of endocrine pancreatic ß-cells to respond to strong insulinotropic stimuli [71]. It is possible that GIPR agonism with tirzepatide is accentuated by the long exposure relative to the effects of acute treatments in older studies [7, 59]. Moreover, glucose lowering with 4 weeks of intensified insulin treatment partially restores the insulinotropic action of GIP (and GLP-1) [72], and this may also occur in response to the glycemic effects of several weeks of tirzepatide treatment. Co-stimulation of GLP-1 receptors may induce a similar phenomenon, especially since there are obvious interactions in the signaling pathways for GIP and GLP-1 [73, 74]. If GIPR agonism induced by tirzepatide treatment is responsible for enhanced ß-cell secretion, it is reasonable to assume a time-dependent process re-establishing GIP as an effective insulin secretagogue even in advanced type 2 diabetes. These possibilities require dedicated mechanistic studies to verify.

**Table 3:** Influence of GLP-1, GIP, their combination, and tirzepatide (dual GIP/GLP-1 receptor co-agonist) on determinants of glycaemic control and body weight in healthy and type 2-diabetic human subjects

Parameter/Population studied	Previous findings regarding short-term exposure to			Current findings with long-term exposure to
	GLP-1	GIP	Combination of GLP-1 and GIP	Tirzepatide (dual GIP/GLP-1 receptor co-agonist)
Insulin secretion				
Healthy subjects	Glucose-dependent stimulation [54, 55]	Glucose-dependent stimulation [55]	Additive glucose-dependent stimulation [55–57]	Not studied
Type 2 diabetes patients	Largely preserved glucose-dependent stimulation [7, 58]	Much-reduced glucose-dependent stimulation (almost no effect) [7, 59]	Not different from effects of GLP-1 alone (negligible effects of GIP) [8]	Stimulated more than with selective GLP-1 RA semaglutide [50] (suggesting some effect of GIPR agonism)
Glucagon secretion				
Healthy subjects	Little effect at moderate hyperglycaemia [7], no effect at hypoglycaemia [60]	Stimulation (dependent on plasma glucose concentrations) [61]	Not studied	Not studied
Type 2 diabetes patients	Suppression at moderate hyperglycaemia [7, 8]	No significant effect at moderate hyperglycaemia [7, 8], stimulation at low plasma glucose [62]	No effect (i.e., the GLP-1-induced suppression is counteracted by concomitant exposure to GIP [8])	Suppression (greater than suppression with selective GLP-1 RA semaglutide) [50] (suggesting some effect of GIPR agonism)
Insulin sensitivity				
Healthy subjects	No acute effects [63]	Not studied	Not studied	Not studied
Type 2 diabetes patients	No acute effects [64]	Not studied	Not studied	Increased with long-term administration (accompanied by substantial weight loss), more than with the selective GLP-1 RA semaglutide [50]
Meal tolerance				
Healthy subjects	Not studied	Not studied	Not studied	Not studied
Type 2 diabetes patients	Improved [65] (mainly through deceleration of gastric emptying)	Slightly worsened [66] (stimulation of glucagon)	Not studied	Improved (more than with selective GLP-1 RA semaglutide) [50]
Glycated haemoglobin (HbA_1c_) Type 2 diabetes patients	Reduced [67]	Not studied	Not studied	Greater reduction in fasting plasma glucose and HbA_1c_ as compared to selective GLP-1 RAs (e.g., dulaglutide [14] or semaglutide [16])
Appetite Healthy or non-diabetic obese subjects	Reduced (robust findings) [68]	Not changed [69]	Not changed [69]	Appetite reduced with tirzepatide and semaglutide (selective GLP-1 RA) to a similar degree [49]
Caloric intake (ad libitum meal) Healthy or non-diabetic obese subjects	Reduced (robust findings) [68, 70]	Not changed [69]	Less reduction compared to GLP-1 alone [69]	Similar reduction compared to selective GLP-1 RA semaglutide [49]
Energy expenditure Healthy or non-diabetic obese subjects	Not changed [69]	Not changed [69]	Not changed [69]	Not studied
Body weight Type 2 diabetes patients	Reduced after 6 weeks of s.c. infusion [67]	Not studied	Not studied	Substantial reduction (see Fig. 3; more than with selective GLP-1 RA semaglutide) [50]

*GLP-1* glucagon-like peptide-1, *GIP* glucose-dependent insulinotropic polypeptide, *GIPR* GIP receptor, *RA* receptor agonist, *HbA*_*1c*_ glycated haemoglobin, fraction A_1c_

In rodents, hypothalamic neurons producing COMT [catechol-O-methyl transferase] and CART [cocaine- and amphetamine-regulated transcript] are involved in the regulation of satiety and energy intake; these neurons also express GIP and/or GLP-1 receptors [75]. Selective stimulation of these neurons reduces food intake [75], but does not potentiate the effects of concomitantly administered selective GLP-1 receptor agonists. Consistent with these observations, both intracerebroventricular and peripheral injections of a GIP receptor agonist reduce energy intake in rodents with diet-induced obesity or diabetes [11], effects that are abolished by genetic deletion of the GIPR. Such studies support a role for GIPR agonism to contribute to weight reduction with dual GIP/GLP-1 receptor co-agonists. However, based on currently available human studies (summarized in Table 3), this mechanism lacks experimental support. To wit, exogenous GIP, given at supraphysiologic doses, did not change ratings of appetite or ad libitum food intake in human subjects [69], whereas, in the same study, exogenous GLP-1 displayed robust effects on both. Surprisingly, combining GLP-1 and GIP led to a lesser suppression of appetite and energy intake compared to GLP-1 alone. Likewise, antagonizing endogenous GIP actions with the specific antagonist GIP (3–30 NH_2_) did not change subjective measures of appetite, satiety, or prospective food consumption after ingesting a mixed meal [57].

Thus, human studies addressing a potential role of GIP in reducing appetite, energy intake, and body weight have produced discordant results from preclinical studies. However, the human studies with GIP done to date have used short-term (maximum 6 days) infusions of native GIP, which may not approximate the effects of tirzepatide with respect to pharmacokinetics, GIP receptor engagement, or interaction with stimulated GLP-1 receptors. Moreover, even in animal experiments, the question remains open as to whether GIP agonism or GIP antagonism is the better way to achieve body weight reduction [76]. In fact, GIP agonism has been proposed to cause desensitization of GIPR signaling and lead to a state of functional antagonism [77]. It is too early to conclude a species difference in this respect, and more science is necessary to align these contrasting findings and to clarify the role of GIP in the regulation of body weight in human subjects in particular.

### Upcoming indications beyond type 2 diabetes: obesity (without diabetes) and non-alcoholic fatty liver disease

The dual GIP/GLP-1 receptor co-agonist combining agonism at both incretin hormones’ receptors (“twincretin”) was originally been designed as a novel treatment for type 2 diabetes. The highly effective reduction in body weight (Fig. 3) observed in type 2-diabetic patients with tirzepatide has shown the obvious potential in the treatment of obesity in the absence of type 2 diabetes. Recently, a dedicated obesity study has been published, which in quantitative terms shows even greater weight loss in the absence of diabetes. Jastreboff et al. reported reductions of 15 to 20.9% of initial body weight with 5 to 15 mg tirzepatide per week [78]. This was accompanied by changes in waist circumference (by up to 18.5 cm with the highest dose), in systolic (-7.2 mmHg) and diastolic (− 4.8 mmHg) blood pressure, and in lipids (e.g., triglycerides − 24.8 mg/dl; HDL cholesterol + 8 mg/dl; VLDL cholesterol − 24.4 mg/dl). The main adverse events reported were again nausea, diarrhoea, vomiting, and constipation [78]. The greater degree of weight loss in non-diabetic obese subjects may in part be explained by the fact that in diabetic subjects with relatively high baseline HbA_1c_ reducing plasma glucose concentrations triggers a reduction in glucosuria. Yki-Järvinen calculated an approximately 2 kg weight difference for a reduction in HbA_1c_ by 1.0% through the influence on caloric losses via glucosuria [79].

Tirzepatide (compared to insulin *degludec*) has been shown to reduce intrahepatic triglycerides in type 2 diabetes [80], and may offer novel treatment options for patients with fatty liver disease (as previously described for selective GLP-1 RAs [81]). It will be important to understand how much of the effect to reduce steatosis is due to weight loss and whether there are also effects independent of this.

### Outlook

Tirzepatide now is the first peptide dual agonist targeting GIP and GLP-1 receptors approved for the therapy of diabetes in the USA, Europe, and the UAE. Clinical trials employing this once-weekly injected compound impress with unprecedented effectiveness regarding glycaemic control (often resulting in normoglycaemia) and body weight reduction, in quantities that are likely to substantially change the underlying pathogenesis of type 2 diabetes [82] and have been associated with diabetes remission [83, 84]). The magnitude of the effects of tirzepatide on glycemia and weight loss opens a new era in diabetes therapy with the promise that a large percentage of patients can be treated to currently established targets. In addition, the clinical efficacy indicates large changes to diabetic physiology and should allow novel questions regarding pathogenesis to be addressed. For example, what does major weight loss added to excellent glycaemic control mean in terms of disease progression and long-term prognosis? And, what effect does the combination of weight loss and dramatic HbA_1c_ reduction have on micro- and macrovascular complications? The next important step will be more information on the cardiovascular consequences of using tirzepatide in type 2 diabetes. The SURPASS CVOT results are eagerly awaited, since there has not been any previous experience with long-term GIPR agonism in clinical medicine. GIP has been described to have anti-atherosclerotic and other beneficial cardiovascular effects [85, 86], but some findings (e.g., stimulation of endothelin-1 in endothelial cells) raise concerns that have not been fully explored [85]. Further, elevated plasma concentrations of GIP in human subjects have been found to be associated with increased carotid intima-media thickness [87] and an increased risk for cardiovascular events and mortality [88]. However, two recent publications examining effects of genetically predicted GIP plasma concentrations on cardiovascular risk factors come to an opposite conclusion and rather suggest beneficial effects of higher exposure to circulating GIP [89, 90]. Thus, probing the cardiovascular effects of tirzepatide is answering a question for which we do not have the answer beforehand, even given the favorable preliminary cardiovascular preliminary analysis [20]. Certainly, this will be an opportunity to learn whether the hitherto unmatched reduction in HbA_1c_ and body weight observed with tirzepatide translates into additional cardiovascular benefits compared to the selective GLP-1 RA dulaglutide.

Further, dual agonism is being tested for various combinations of receptors, e.g., GLP-1R/glucagon-R, GLP-1R/amylin-R, GLP-1R/NPYR (peptide YY binds to NPY receptors) [91, 92]. Some results of clinical studies have been published and promise effect sizes not usually observed with selective GLP-1 RAs alone [93, 94]. Thus, tirzepatide may be the first dual agonist to be approved, but more compounds will follow, and may perhaps present further advances in the treatment of type 2 diabetes and obesity and in associated conditions.

## Supplementary Information

**Additional file 1: Figure S1**. Meta-analysis of results reported from SURPASS-1 to -5 clinical trials regarding reductions in HbA_1c_, fasting plasma glucose concentrations, and body weight reductions (all vs. baseline). Efficiacy results were analysed based on the reported efficacy estimand (calculated from data gathered while patients received their assigned randomized treatment, excluding data after discontinuing the allotted medication or after adding rescue medication. Results obtained with each dose (5, 10, or 15 mg per week) were pooled by calculating weighted mean values and pooled standard deviations. Statistical analysis: Repeated-measures analysis of variance (p-value, for the comparison of all four study arms) and *post hoc* Duncan’s tests to locate statistically significant differences (if overall analysis had indicated a p-value < 0.05) between any two doses of tirzepatide. Heterogeneity: 3 doses of tirzepatide and 5 studies indicate 14 degrees of freedom, q was 0.02, 28.3, 0.91 (not significant, p < 0.0001, and non-significant, respectively) and I^2^ was  0, 13.1, and 0 %, respectively, for HbA_1c_, fasting plasma glucose, and body weight reductins vs. baseline. Overall, the degree of heterogenity for these analyses can be considered negligible or low. **Figure S2.** Meta-analysis of results reported from SURPASS-1 to -5 clinical trials regarding achievement of HbA_1c_ targets. Results obtained with each dose (5, 10, or 15 mg per week) were pooled by calculating weighted mean values and pooled standard deviations. Statistical analysis: χ^2^ test (p-value, for the comparison of all four study arms) and *post hoc* Fisher’s exact tests to locate statistically significant differences (if overall analysis had indicated a p-value < 0.05) between any two doses of tirzepatide. **Figure S3.** Meta-analysis of results reported from SURPASS-1 to -5 clinical trials regarding discontinuation of study medications (A. overall; B. due to gastro-intestinal adverse events) and gastro-intestinal adverse events (patients reporting at least one episode of C. nausea; D. vomiting; E. diarrhoea). Results obtained with each dose (5, 10, or 15 mg per week) were pooled by calculating weighted mean values and pooled standard deviations. Statistical analysis: Statistical analysis: χ^2^ test (p-value, for the comparison of all four study arms) and *post hoc* Fisher’s exact tests to locate statistically significant differences (if overall analysis had indicated a p-value < 0.05) between any two doses of tirzepatide.

## Data Availability

The manuscript entirely reports published data. Spreadsheets with data as compiled for the present manuscript can be made available on reasonable request to the corresponding author.
