# Supplementary material for: Tirzepatide, a dual GIP/GLP-1 receptor co-agonist for the treatment of type 2 diabetes with unmatched effectiveness regrading glycaemic control and body weight reduction
Source: Cardiovasc Diabetol. 2022 Sep 1;21:169. doi: 10.1186/s12933-022-01604-7 (PMC9438179; doi:10.1186/s12933-022-01604-7)
Supplement: Supplementary file 1 — Additional file 1: Figure S1. Meta-analysis of results reported from SURPASS-1 to -5 clinical trials regarding reductions in HbA1c, fasting plasma glucose concentrations, and body weight reductions (all vs. baseline). Efficiacy results were analysed based on the reported efficacy estimand (calculated from data gathered while patients received their assigned randomized treatment, excluding data after discontinuing the allotted medication or after adding rescue medication. Results obtained with each dose (5, 10, or 15 mg per week) were pooled by calculating weighted mean values and pooled standard deviations. Statistical analysis: Repeated-measures analysis of variance (p-value, for the comparison of all four study arms) and post hoc Duncan’s tests to locate statistically significant differences (if overall analysis had indicated a p-value < 0.05) between any two doses of tirzepatide. Heterogeneity: 3 doses of tirzepatide and 5 studies indicate 14 degrees of freedom, q was 0.02, 28.3, 0.91 (not significant, p < 0.0001, and non-significant, respectively) and I2 was 0, 13.1, and 0 %, respectively, for HbA1c, fasting plasma glucose, and body weight reductins vs. baseline. Overall, the degree of heterogenity for these analyses can be considered negligible or low. Figure S2. Meta-analysis of results reported from SURPASS-1 to -5 clinical trials regarding achievement of HbA1c targets. Results obtained with each dose (5, 10, or 15 mg per week) were pooled by calculating weighted mean values and pooled standard deviations. Statistical analysis: χ2 test (p-value, for the comparison of all four study arms) and post hoc Fisher’s exact tests to locate statistically significant differences (if overall analysis had indicated a p-value < 0.05) between any two doses of tirzepatide. Figure S3. Meta-analysis of results reported from SURPASS-1 to -5 clinical trials regarding discontinuation of study medications (A. overall; B. due to gastro-intestinal adverse events) and gastro-intestin [file 12933_2022_1604_MOESM1_ESM.docx]

**Online Supplementary Material**

**Tirzepatide, a Dual GIP/GLP-1 Receptor Co-Agonist for the Treatment of Type 2 Diabetes with Unmatched Effectiveness Regrading Glycaemic Control and Body Weight Reduction**

Michael A. Nauck, M.D. ^1^, David A. D‘Alessio, M.D. ^2^

^1^ Diabetes, Endocrinology and Metabolism Section, Medical Department I, Katholisches Klinikum Bochum gGmbH, St. Josef-Hospital, Ruhr-University Bochum, Bochum, Germany

^2^ Division of Endocrinology and Metabolism, Duke Molecular Physiology Institute

Duke University, Durham, NC 27701, USA

**Supplementary Figure 1.** **Meta-analysis of results reported from SURPASS-1 to -5 clinical trials regarding reductions in HbA_1c_, fasting plasma glucose concentrations, and body weight reductions (all vs. baseline).** Efficiacy results were analysed based on the reported efficacy estimand (calculated from data gathered while patients received their assigned randomized treatment, excluding data after discontinuing the allotted medication or after adding rescue medication. Results obtained with each dose (5, 10, or 15 mg per week) were pooled by calculating weighted mean values and pooled standard deviations. Statistical analysis: Repeated-measures analysis of variance (p-value, for the comparison of all four study arms) and *post hoc* Duncan’s tests to locate statistically significant differences (if overall analysis had indicated a p-value < 0.05) between any two doses of tirzepatide. Heterogeneity: 3 doses of tirzepatide and 5 studies indicate 14 degrees of freedom, q was 0.02, 28.3, 0.91 (not significant, p < 0.001, and not significant, respectively), and I^2^ was 0, 13.1, and 0 %, respectively, for HbA_1c_, fasting plasma glucose, and body weight reductions vs. baseline. Overall, the degree of heterogeneity for these analyses can be considered negligible or low.

**Supplementary Figure 2.** **Meta-analysis of results reported from SURPASS-1 to -5 clinical trials regarding achievement of HbA_1c_ targets.** Results obtained with each dose (5, 10, or 15 mg per week) were pooled by calculating weighted mean values and pooled standard deviations. Statistical analysis: χ^2^ test (p-value, for the comparison of all four study arms) and *post hoc* Fisher’s exact tests to locate statistically significant differences (if overall analysis had indicated a p-value < 0.05) between any two doses of tirzepatide.

**Supplementary Figure 3.** **Meta-analysis of results reported from SURPASS-1 to -5 clinical trials regarding discontinuation of study medications (A. overall; B. due to gastro-intestinal adverse events) and gastro-intestinal adverse events (patients reporting at least one episode of C. nausea; D. vomiting; E. diarrhoea).** Results obtained with each dose (5, 10, or 15 mg per week) were pooled by calculating weighted mean values and pooled standard deviations. Statistical analysis: Statistical analysis: χ^2^ test (p-value, for the comparison of all four study arms) and *post hoc* Fisher’s exact tests to locate statistically significant differences (if overall analysis had indicated a p-value < 0.05) between any two doses of tirzepatide.
